# An innovative combination of Box-Behnken design and ecofriendly approaches for the simultaneous determination of aspirin, clopidogrel, atorvastatin and rosuvastatin in their fixed-dose combination tablets

**DOI:** 10.1186/s13065-023-01079-x

**Published:** 2023-11-24

**Authors:** Eman A. Mostafa, Mohamed K. El‐Ashrey, Sally Tarek Mahmoud

**Affiliations:** 1 Pharmaceutical Chemistry Department, Faculty of Pharmacy, Cairo University, Kasr El-Aini St., Cairo, 11562 Egypt; 2 Medicinal Chemistry Department, Faculty of Pharmacy, King Salman International University, Ras-Sedr, South Sinai Egypt

**Keywords:** Aspirin, Clopidogrel, Atorvastatin, Rosuvastatin, HPLC–DAD, Box-Behnken design, Green profile

## Abstract

Three-levels Box-Behnken design was used in the experimental design approach for the optimization of chromatographic parameters to achieve the optimum resolution and sharp peak shape within a reasonable run time. A method that is sensitive, reliable, and selective was constructed and validated for the simultaneous measurement of a combination therapy that contains blood-thinning and cholesterol-lowering compounds. The four cited drugs namely, aspirin (ASP), clopidogrel (CLP), atorvastatin (ATV) and rosuvastatin (ROS) were estimated in bulk and in pharmaceutical dosage forms in line with International Council for Harmonization guidelines. The separation was done utilizing Kinetex 2.6 C18 column (100 mm, 4.6 mm, 5 m) and RP-HPLC with diode array detector. The separation of the cited drugs and the degradation product of ASP was achieved with mobile phase composed of acetonitrile: KH_2_PO_4_ buffer in a gradient mode with pH 3.2 at room temperature. The four drugs were linear over the concentration range (0.05–50 µg/mL). The technique is feasible to be used in quality control laboratories. To picture the green profile of the developed method, four greenness assessment tools were applied. National environmental methods index (NEMI), analytical eco-scale assessment (ESA), green analytical procedure index (GAPI) and analytical greenness metric (AGREE) are the most widely used metrics. They were employed to evaluate the greenness profile of the proposed method and to perform a detailed greenness comparison between the developed method and some of the reported methods for the determination of the investigated drugs. The developed method was found to be relatively green with 0.54 AGREE score.

## Introduction

**Fig. 1:**
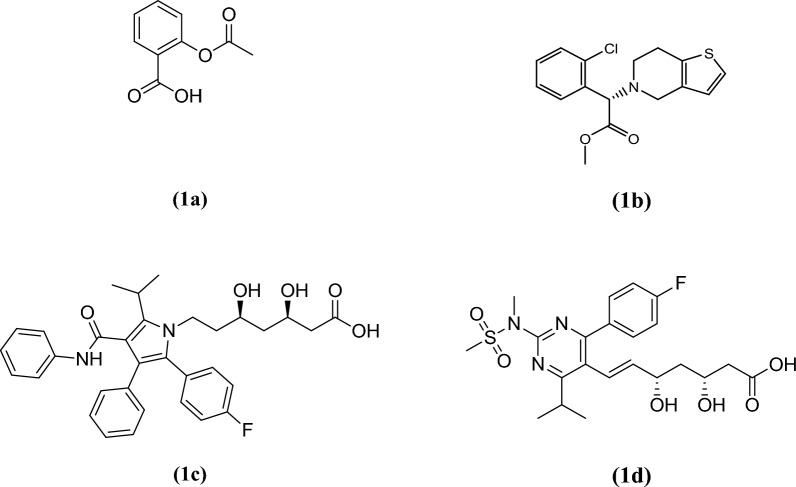
Chemical structures of aspirin (**1a**), clopidogrel (**1b**), atorvastatin (**1c**) and rosuvastatin (**1d**)

Acute coronary syndrome (ACS) is a term that refers to a group of disorders coupled with sudden, reduced blood flow to the heart. It significantly contributes to cardiovascular mortality and morbidity worldwide. And it is constantly associated with rupture of an atherosclerotic plaque and partial or complete clotting of the infarct-related artery [1]. Thrombus formation and platelet aggregation play a crucial role in the initiation and development of major problems of acute coronary syndromes [2]. Moreover, the major risk factor for fatal cardiovascular disease is high blood cholesterol. Therefore, Dual antiplatelet therapy (DAPT) that along with hypolipemic drugs provided evidence to be a good therapeutic option for patients with ACS [3]. As antiplatelet therapy and antithrombotic therapy have been proven favorable clinical outcomes, and a reduction in the frequency of major cardiac events [4]. Hence, several advantages have been demonstrated for the use of fixed dose combination (FDC) in cardiovascular diseases such as reduction in adverse effects, cost, improved patient compliance. Multidrug therapy with aspirin, clopidogrel and atorvastatin or rosuvastatin (Fig. 1) have been proposed as a treatment method to lower the risk of cardiovascular disease [5].

**Fig. 2:**
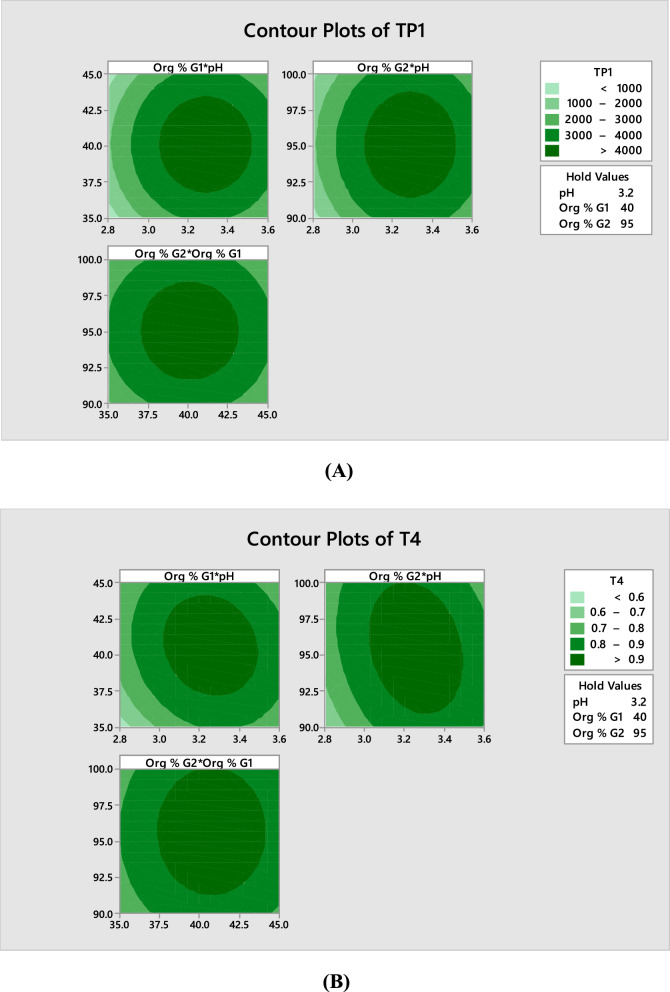
Contour plots showing the effect of the selected factors on TP1 (theoretical plates of peak 1) (**A**) and T4 (tailing factor of peak 4) (**B**)

**Fig. 3:**
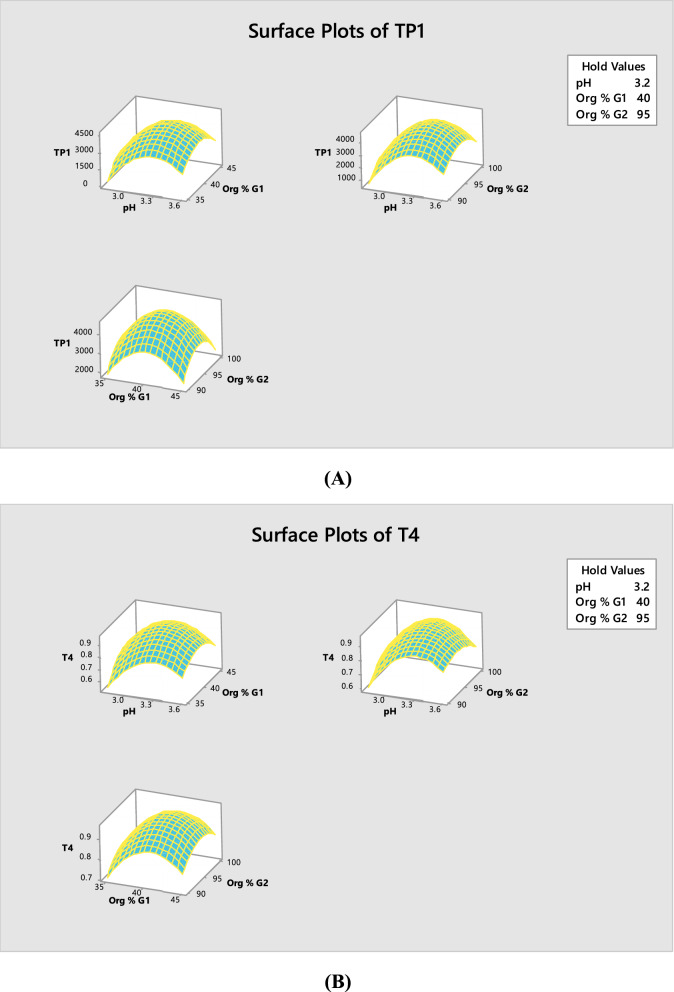
Response surface plots showing the effect of the selected factors on TP1 (theoretical plates of peak 1) (**A**) and T4 (tailing factor of peak 4) (**B**)

**Fig. 4:**
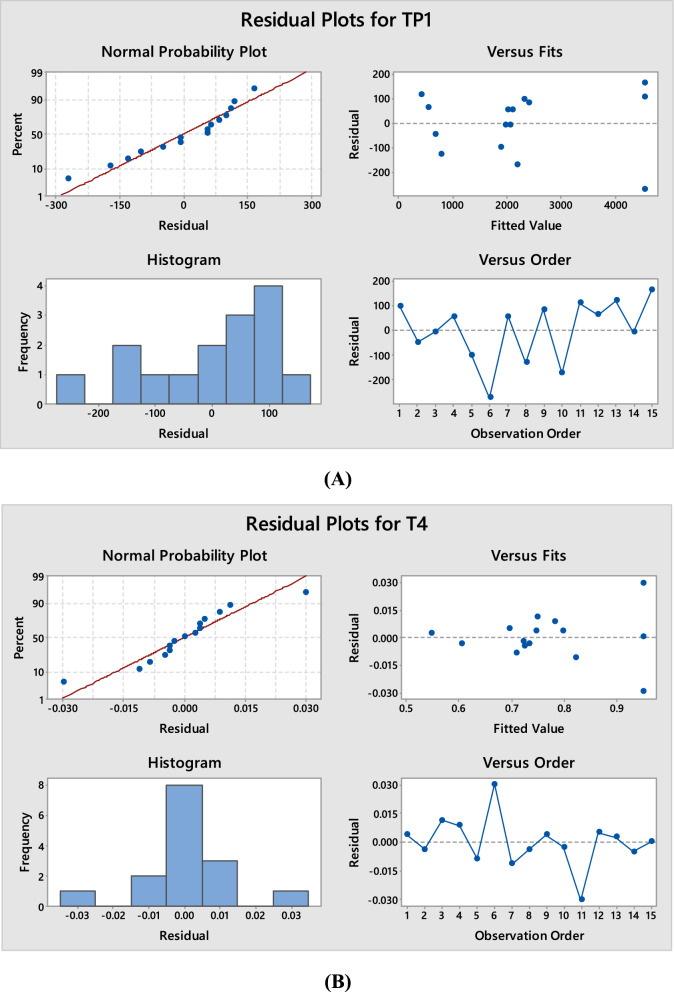
Normal probability plot, histogram, versus fits and versus order for TP1 (theoretical plates of peak 1) (**A**) and T4 (tailing factor of peak 4) (**B**)

Aspirin (ASP) is known chemically as [2-acetyloxybenzoic acid] (Fig. 1a). It is proved to inhibit platelet aggregation as it interferes with thromboxane A2 in platelets, This is due to the fact that thromboxane A2 is a crucial lipid involved in platelet aggregation, which might result in clot formation and increase the risk of heart attack or stroke in the future [6], besides its anti-inflammatory, analgesic and antipyretic actions. In aqueous solution, ASP is known to undergo decomposition by hydrolysis into salicylic acid (SA), and it is reported that the decomposition reaction is promoted at high temperatures. Because ASP is rapidly de-acetylated by esterase in human plasma, much of ASP’s bioactivity can be attributed to its primary metabolite, SA. Clopidogrel (CLP), its chemical name is [methyl (2*S*)-2-(2-chlorophenyl)-2-(6,7-dihydro-4*H*-thieno[3,2-c] pyridin-5-yl) acetate] (Fig. 2a). It is an antiplatelet agent, that inhibits adenosine diphosphate (ADP) binding selectively to its platelet receptor in addition to blocking the succeeding ADP-mediated triggering of the glycoprotein GPIIb/IIIa complex, thus inhibiting platelet aggregation [7]. It has been proven to prevent myocardial infarction, ischemic stroke and vascular disease [8]. Atorvastatin (ATV) is known chemically as [(3R,5R)-7-[2-(4-fluorophenyl)-3-phenyl-4-(phenylcarbamoyl)-5-propan-2-ylpyrrol-1-yl]-3,5-dihydroxyheptanoic acid] (Fig. 3a), while the chemical name of Rosuvastatin (ROS) is [(*E*,3*R*,5*S*)-7-[4-(4-fluorophenyl)-2-[methyl(methylsulfonyl)amino]-6-propan-2-ylpyrimidin-5-yl]-3,5-dihydroxyhept-6-enoic acid] (Fig. 4a). Statins are inhibitors of HMG-CoA reductase, they are the most efficient agents for lowering plasma cholesterol and used for the treatment of hypercholesterolemia [9]. Consequently, Statins considerably reduce the frequency of coronary events, being the most efficient hypolipidemic substances that have lowered the death rate in individuals with coronary artery disease [10]. Based on the importance of those drugs combination, there is a great need for developing analytical procedures capable of their simultaneous determination in FDC tablets.

Literature survey revealed that different quantitative methods have been reported for the analysis of ASP, CLP, and ATV mixture in their dosage form utilizing LC-UV [11–15], UV spectrophotometry [16, 17], HPTLC [18] and in biological fluids [19]. While the mixture of ASP, CLP, and ROS was quantified using UV spectrophotometry [20, 21] and LC-UV [22]. In addition, CLP and ROS were simultaneously determined using LC-UV [23], besides ASP and CLP were determined together with other antiplatelet drugs using LC-UV [24]. In addition, ASP was determined in presence of ASP degradation using LC-UV [25–27]. A simple comparison between the proposed method and some of previously reported methods for the analysis of ASP in presence of its degradation product, salicylic acid (SA) could be summarized as follow; The proposed method was superior in terms of it has a wide ASP linearity range of 0.05–50 μg/mL while linearity was in the range of 1–150 μg/mL for ASP, 1–25 μg/mL for SA [25] and 34.8–97.1 μg/mL for ASP, 0.3–3.4 μg/mL for SA [27] and 17.42–87.10 for ASP, 8.33–41.67 for SA [26] Also in terms of retention time of SA, ASP were 3.51 min, 4.77 min [25] and 3.3 min, 1.5 min [27] and 10.65 min, 5.61 min [26] and 1.201 min, 2.021 min in the current study, respectively.

Green analytical chemistry (GAC) has become the focus of attention since 2000 in the analytical chemist’s community, as it is the only way to attempt to preserve the environment. GAC target is to protect humans and the environment from the severe damage they are exposed to as a result of using analytical methods that consume chemicals, produce waste and utilize devices that cause deep damage to the ecosystem [28, 29]. In GAC, in order to create a greener environment, the green aspects should be taken into consideration from the initial stages of method development. In addition, a well-established practice should be designed to ensure the reduction or disposal of hazardous materials that are used in or produced by this method to provide a safer method for the environment [30–32]. Approved GAC principles and recommendations are the cornerstones of balancing effective analysis and safe procedures. The GAC's fundamental principles were adopted and published [33, 34]. However, there was a lack of published standard tools or techniques for greenness assessment of the developed analytical methods to suggest if a particular analytical procedure is accepted as green one or not. Moreover, the evaluation tools ought to be efficiently compared and involved as a guideline in the development and validation of new eco-friendly analytical methods [29, 35]. National environmental methods index (NEMI) [36], eco-scale assessment (ESA) [37], green analytical procedure index (GAPI) [38] and analytical greenness metric (AGREE) [39] are the most applied greenness assessment tools. It is recommended to combine the four methods upon assessing and/or comparing the greenness of analytical method (s) to get a deeper view about the green profile of the assessed methods [40, 41]. Moreover, Different analytical parameters need to be monitored in-depth and the interaction between them should be investigated closely to ensure that the method efficiency is not affected by applying GAC principles. This could be offered through adopting the design of experiment.

Design of experiment (DOE) is extremely crucial as it is considered as an efficient optimization procedure because it takes into account the interaction between critical factors affecting the chromatographic separation. Moreover, it offers great advantages over one variable at a time (OVAT) procedure as it requires the fewest possible experiments during the optimization process and yields useful information regarding the interactions between the experimental parameters [42–46].

And to the best of our knowledge after searching the literature, there is no green RP-HPLC method with an efficient optimization procedure was established for the simultaneous determination of ASP, CLP, ATV, and ROS. Based on the aforementioned considerations, the aim of the current study is to combine the benefits of utilizing the most relevant DOE methodology, Box–Behnken optimization design and greenness assessment approaches for simultaneous determination of ASP, CLP, ATV and ROS in the presence of ASP degradation (salicylic acid) in bulk and in their combined dosage forms.

## Experimental

### Instruments and software

(Shimadzu instrument, Japan) chromatographic system was equipped with a mixer, a vacuum degasser, a gradient pump and Diode array detector. Separation and quantitation were achieved on Kinetex 2.6 µ C_18_ column 100 Å (100 mm, 4.6 mm, 5 µm). A (Power Sonic 405, Korea, HumanLab) sonicator was utilized. pH meter (Jenway, 3505, Essex, UK.) for pH measurements. And membrane filters (Sartorius Stedim Biotech GmbH, 0.45 µm, Goettingen, Germany) were utilized for filtration of mobile phase. Version 17 Minitab, Statistical Software; Minitab, Inc.: State College, PA, USA, 2014.

### Materials and reagents

ASP, CLP, ATV and ROS standards were obtained from the National Organization of Drug Control and Research (NODCAR), Egypt (Their purity certified to contain 99.80%, 99.75%, 99.93% and, 99.85%, respectively). Rosutor gold tablets which nominally contain 10 mg ROS, 75 mg ASP and 75 mg CLP per one tablet were purchased. In addition, Ecosprin tablets contain 10 mg ATV, 75 mg ASP and 75 mg CLP per one tablet. Bi-distilled water was produced on (Aquatron Water Still, A4000D, UK). (HPLC grade) Acetonitrile was obtained from Sigma-Aldrich, Germany. Extra pure potassium dihydrogen phosphate was bought from Lobachemie, India and utilized for buffer preparation. Salicylic acid was obtained from El-Nasr Pharmaceutical Chemicals Co., Al-Kalubia, Egypt.

### Stock solutions

An accurate weight (50 mg) of ASP, CLP, ATV and ROS was transferred separately in 100 mL volumetric flasks and dissolved in 1 mL methanol, then the volume was completed with distilled water in order to obtain a stock solution of (0.5 mg/mL). While salicylic acid (ASP degradation) stock solution (1 mg/mL) was prepared by dissolving 5 mg of salicylic acid in methanol and completing the volume to the mark in 50 mL volumetric flask.

### Sample preparation

Twenty tablets of Rosutor and Ecosprin were exactly weighed then ground to fine powder separately. An amount of each powder equal to one tablet Rosutor contained (10 mg ROS, 75 mg ASP and 75 mg CLP) and one tablet Ecosprin contained (10 mg ATV, 75 mg ASP and 75 mg CLP) were transferred into a 100 mL measuring flask, 5 mL methanol was added, and the flasks were sonicated for 10 min. To obtain a sample stock solution, the volume was filled to the mark with distilled water (100 µg/mL ROS, 750 µg/mL ASP and 750 µg/mL CLP) for Rosutor gold tablet and (100 µg/mL ATV, 750 µg/mL ASP and 750 µg/mL CLP) for Ecosprin tablet, respectively. Whatman filter paper was used to filter the resulting sample stock solutions, the initial few milliliters are discarded. Aliquots from the produced stock solution were transferred to a series of 10 mL measuring flasks and the volumes were completed to the mark with the distilled water for the determination the cited drugs.

### Design of experiment for chromatographic conditions optimization

To optimize the key parameters impacting HPLC separation, three levels Box-Behnken design with three center points was applied to study the effects of pH, the mobile phase organic ratio in the first stage of gradient elution (Org% G1) and organic ratio in the mobile phase in the second stage of gradient elution (Org% G2) on theoretical plates of first peak (TP1) and tailing factor of peak 4 (T4). Table 1, Describes the composition of fifteen experimental runs that were designed and carried out by injecting studied drugs mixture. To analyze the data, statistical software (Minitab® 17) was employed. The model obtained was described by a second-order mathematical equation which takes into account the individual, interactive and quadratic terms. For each response, contour plots and response surface plots were created. And to ensure the model's efficacy, Plots of residuals and a lack of fit test with the analysis of variance (ANOVA) model were carried out [47, 48].

**Table 1:** Experimental matrix and experimental plan of Box-Behnken design

Experimental run	X1	X2	X3	pH	Organic ratio G1 (%)^a^	Organic ratio G2 (%)^b^
1	1	0	− 1	3.6	40	90
2	− 1	0	− 1	2.8	40	90
3	0	− 1	1	3.2	35	100
4	0	1	− 1	3.2	45	90
5	0	− 1	− 1	3.2	35	90
6	0	0	0	3.2	40	95
7	0	1	1	3.2	45	100
8	− 1	0	1	2.8	40	100
9	1	0	1	3.6	40	100
10	1	1	0	3.6	45	95
11	0	0	0	3.2	40	95
12	− 1	1	0	2.8	45	95
13	− 1	− 1	0	2.8	35	95
14	1	− 1	0	3.6	35	95
15	0	0	0	3.2	40	95

^a^Organic ratio of gradient (1)

^b^Organic ration of gradient (2)

### Chromatographic conditions

The chromatographic separation and quantification were conducted utilizing a stationary phase consisted of Kinetex 2.6 µ C_18_ column 100 Å (100 mm, 4.6 mm, 5 µm). The mobile phase consisted of 80% acetonitrile in distilled water (A) and 20 mM potassium dihydrogen phosphate buffer (pH = 3.2 adjusted with *o*-phosphoric acid) (B) in a gradient mode at a flow rate 1 mL min^−1^. The applied gradient program comprised of 40% A for 5 min, thereafter % A was increased to 95% over 1 min. Mobile phase (A) was then kept at 95% for 2 min. At 8.5 min, the mobile phase was reverted to 40% A in 0.3 min and remained at initial conditions till 10 min. The total run time was 10 min. An ultrasonic bath was used to degas the mobile phase after being filtered via a 0.45 µm membrane filter. Prior to injecting the solutions, the system was equilibrated and saturated with the mobile phase for 30 min. At room temperature, all determinations were conducted. Utilizing UV detection at λ 230 nm, peak area was used to quantify the results.

### Procedure

- (a) Linearity
  Standard stock solutions aliquots that are equal to 0.5–500 µg/mL of ASP, CLP, ATV and ROS were transferred separately into a series of 10 mL measuring flasks. The solutions were completed to volume using distilled water. Each solution was injected in triplicates with a volume of 10 µL. The chromatographic parameters mentioned above were used and the area under the peak (AUP) was plotted against the relevant drug concentration to create calibration curves.
- (b) Assay of laboratory prepared mixtures
  Several aliquots of ASP, CLP and ATV or ASP, CLP and ROS stock solutions were introduced into two different series of 10 mL measuring flasks with aliquots of ASP degradation product and completed to volume with the distilled water to achieve concentrations between (1.5–50 µg/mL) for ASP and CLP. While ATV or ROS concentrations were in the range of (0.1–10 µg/mL). Then ASP degradation product was added in concentration range (0.75–25 µg/mL). The method was carried out as mentioned in the “Linearity" section and the concentrations were calculated applying the corresponding regression equations.
- (c) Assay of aspirin, clopidogrel and atorvastatin or rosuvastatin in pharmaceutical preparations
  The procedure mentioned above was repeated for the simultaneous determination of cited drugs in Rosutor gold and Ecosprin tablets, after being serially diluted, the sample solutions were then injected in triplicates. The corresponding regression equations were used to compute the concentrations.

## Results and discussion

### Optimization of the chromatographic conditions using Box-Behnken design

DOE is a sequential process used to design and analyze experiments as it works for the identification of important factors and discovery of the factor settings that produce the optimal response. For the method optimization with minimal effort, resources, and time; DOE was applied [43, 49, 50]. In addition, the use of DOE is in favor of GAC as it will minimize the total number of experimental runs needed to reach the best separation conditions and this will reduce waste as well. Box-Behnken Design was utilized to optimize and assess the main effects, quadratic effects, and interaction effects of independent parameters on the interested responses, i.e., BBD considers the linear and quadratic effects, as well as interaction effects among the variables under investigation.

Three levels Box-Behnken design with three center points was used. The levels of each factor are displayed in (Table 1) where the response measurement at each factor’s center point (level zero) was carried out three times to assess the experimental error, whereas all other experimental runs were carried out randomly without replication. Several responses were investigated and their impact on the chromatographic separation was studied. TP1 and T4 were found to be the most effective responses in the chromatographic separation in order to achieve the optimization of the developed method, where TP1: is the theoretical plates number of ASP peak and T4: is the tailing factor of CLP peak, respectively. Therefore, TP1 and T4 were chosen to build the models. The second order polynomial equations describing the models were calculated and found to be:

1 $$ \begin{aligned} {\mathbf{TP1}} &=  - {625217 } + {75694}\, {\text{pH }} + {4462} {\text{Org}} \% \, {\text{G1 }} \\ &\quad+ {8745}\, {\text{Org}} \%\, {\text{G2 }} - {115}0{7} \,{\text{pH }}*\,{\text{ pH}} \\&\quad - {55}.{62}\, {\text{Org}} \% \,{\text{G1 }}*\,{\text{ Org}} \%\, {\text{G1 }} \\ &\quad- {45}.{98} \,{\text{Org}} \% \,{\text{G2 }}*\,{\text{ Org}} \% \,{\text{G2}} \\ \end{aligned} $$

2 $$ \begin{aligned} {\mathbf{T4}} &=  - {51}.{95 } + {9}.{466}\, {\text{pH }} + 0.{4392}\, {\text{Org}} \%\, {\text{G1 }}\\ &\quad + 0.{5985} \,{\text{Org}} \% \,{\text{G2 }} - {1}.00{78} \,{\text{pH}}*\,{\text{pH}} \\ & \quad - 0.00{465}0 \,{\text{Org}} \% \,{\text{G1}}*\,{\text{ Org}} \% \,{\text{G1 }} \\ &\quad- 0.00{275}0 \,{\text{Org}} \% \,{\text{G2}}*\,{\text{Org}} \%\, {\text{G2 }} \\ &\quad- 0.0{1875} \,{\text{pH}}*\,{\text{Org}} \% \,{\text{G1}} \\ & \quad - 0.0{225}0 \,{\text{pH}}*\,{\text{Org}} \% \,{\text{G2}} \\ \end{aligned} $$

where **TP1**: is the theoretical plates number of ASP peak and **T4**: is the tailing factor of CLP peak, respectively.

The present model considers linear effects, quadratic effects, and interactions between the studied factors. A stepwise backward elimination technique was chosen to decrease the number of insignificant terms.

For TP1 and T4; the maximum count of theoretical plates and the highest symmetry of CLP peak is obtained using a pH = 3.2, Org% G1 = 40% and Org% G2 = 95%.

### Effect of factors

Equation (1) demonstrates that TP1 is directly proportional to pH, Org % G1 and Org % G2 while the quadratic terms of pH, Org % G1 and Org % G2 are negative. Although that Eq. (2) reveals that T4 is directly proportional to pH, Org % G1 and Org % G2 but the coefficient factor is much smaller than that of Eq. (1) which means that those factors have a much greater impact on TP1 than T4. In addition, all the quadratic terms of pH, Org % G1 and Org % G2 and the interaction terms between pH and Org % G1, pH and Org % G2 are negative in Eq. (2). The individual effects of pH, Org % G1 and Org % G2 are positive, and their quadratic effects are negative at the same time, showing that both TP1 and T4 increase as the level of the factors increases until a critical point, at which any further rise results in a fall in the response.

Figures 2 and 3 displayed the two polynomial equations’ graphical representation of two-dimensional contour plots and three-dimensional response surface plots illustrating the effect of the three factors on the equation output. The contour plots’ curvature denotes the factors with non-linear effects on TP1 and T4. These figures assist the response prediction at any area of the experimental domain [43]. The maximum response is either close to the center of the contours or the top of the mountain, respectively. For ASP peak, maximum theoretical plates are obtained upon using buffer pH 3.2, Org % G1 40% and Org % G2 95%, Also for CLP peak, the optimum tailing factor value corresponds to buffer pH 3.3, Org % G1 40% and Org % G2 95%.

### Statistical analysis of the model

Utilizing the statistical program Minitab® 17, experiment findings were statistically examined. The ANOVA test was used to validate the models. The regression coefficients and their associated *p* values have demonstrated which of the factors significantly affects the response. (Table 2) demonstrates ANOVA results that prove that the significant factor is the pH for TP1 response and pH, Org % G1 and Org % G2 for T4 response given that their* p* values are below 0.05. R^2^, adjusted R^2^ and predicted R^2^ assess the models fitting and predictive capacities. (Table 3) shows that the R^2^, adjusted R^2^ and predicted R^2^ are very near to each other, and they are larger than 0.9 indicating that the models fit the data well and have a high degree of predictive capacity for future observations and optimization studies. As shown in (Table 2), the lack-of-fit calculated *p*-value for both TP1 and T4 responses is greater than 0.05; proving that the models accurately reflect the experimental results, at 95% confidence level.

**Table 2:** ANOVA results of the models

Source of variation	TP1^a^	T4^b^
	*p*-value	*p*-value
Regression model	0.000	0.000
Constant	0.000	0.001
pH	0.000	0.000
Organic ratio % G1	0.309	0.002
Organic ratio % G2	0.417	0.028
(pH)^2^	0.000	0.000
(Organic ratio % G1)^2^	0.000	0.000
(Organic ratio % G2)^2^	0.000	0.001
(pH*Organic ratio % G1)	–	0.009
(pH*Organic ratio % G2)	–	0.004
Lack-of-Fit	0.896	0.949

^a^Theoretical plates of peak number (1) corresponding to Aspirin

^b^Tailing factor of peak number (4) corresponding to Clopidogrel

**Table 3:** Models fitting results

Model term	TP1^a^	T4^b^
R^2^	0.9921	0.9881
Adjusted R^2^	0.9862	0.9723
Predicted R^2^	0.9758	0.9613

^a^Theoretical plates of peak number (1) corresponding to Aspirin

^b^Tailing factor of peak number (4) corresponding to Clopidogrel

### Residual analysis

Figure 4 displays the analysis response residual plots. The residuals often follow a straight line in normal probability plots, indicating that the normal distribution of the errors, supporting the idea that the models fit the data. The histogram plots clearly show a pattern with normal distribution, proving that the residuals are distributed normally. The assumption that the residuals are independent from one another are supported by the residuals versus order plot. In versus fit and versus order plots, the fact that residuals are randomly distributed around the zero indicates that the error terms are unrelated to one another.

### The optimum separation conditions calculation

**Fig. 5:**
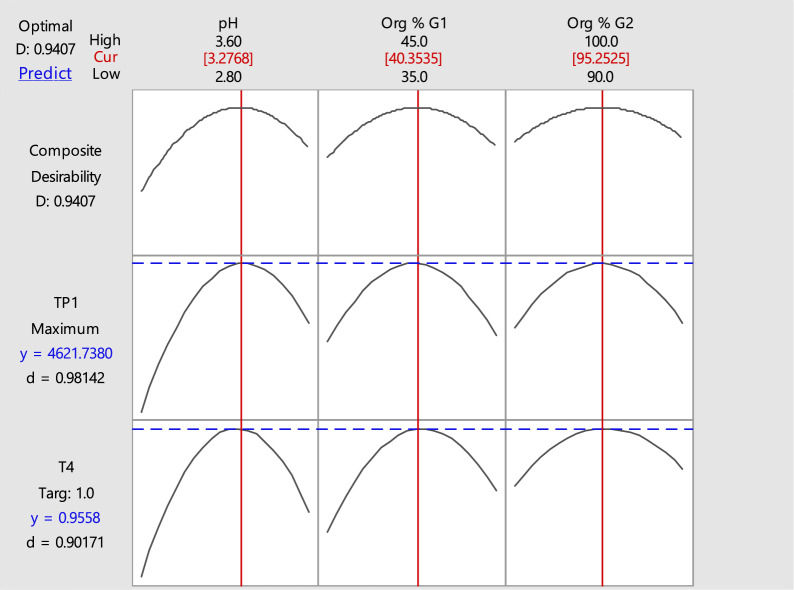
Response optimization showing the calculated desirability factor of the used responses

Using the response optimizer tool and the obtained final models, the optimal conditions that simultaneously yield the best values of TP1 and T4 are derived. The desired goal was maximizing TP1 and achieving the best peak symmetry by targeting T4 = 1, the response optimizer computes the optimum solution to achieve that goal and generates the optimization plot, Fig. 5.

**Fig. 6:**
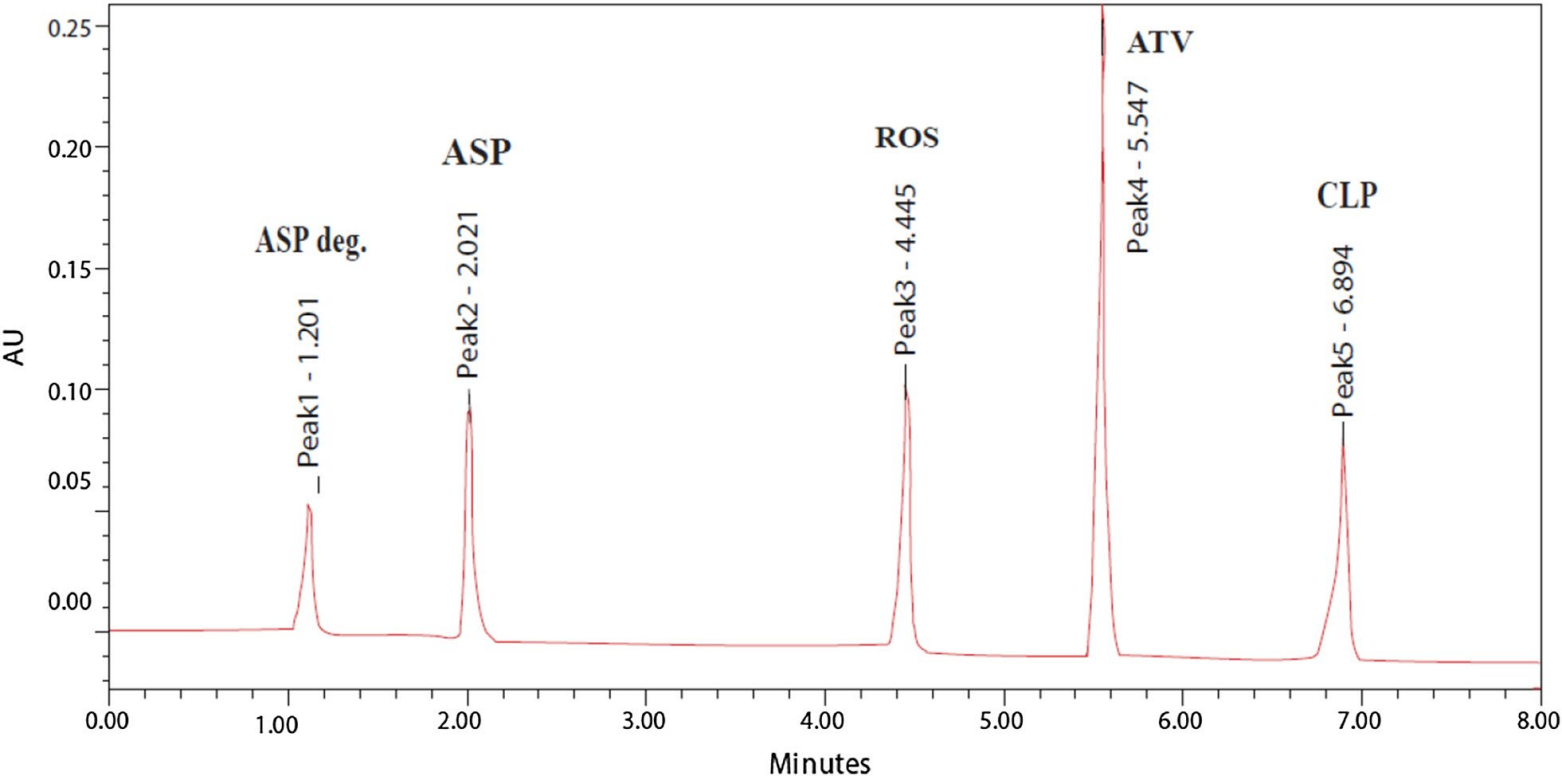
HPLC chromatogram of a laboratory prepared mixture of ASP and ASP degradation (5 ASP μg/mL), (10 μg/mL), ROS (10 μg/mL), ATV (10 μg/mL) and CLP (10 μg/mL)

Utilizing the prior design, symmetric peaks and an acceptable separation were observed upon using a gradient elution of mobile phase consisting of 80% acetonitrile in distilled water (A) and 20 mM potassium dihydrogen phosphate buffer (pH = 3.2 adjusted with *o*-phosphoric acid) (B) at flow rate of 1 mL min^−1^ at ambient temperature. The detection wavelength was selected as (230 nm). The retention times of ASP degradation, ASP, ROS, ATV and CLP were found to be 1.201, 2.021, 4.445, 5.547 and 6.894 min, respectively, Fig. 6.

### Method validation

**Fig. 7:**
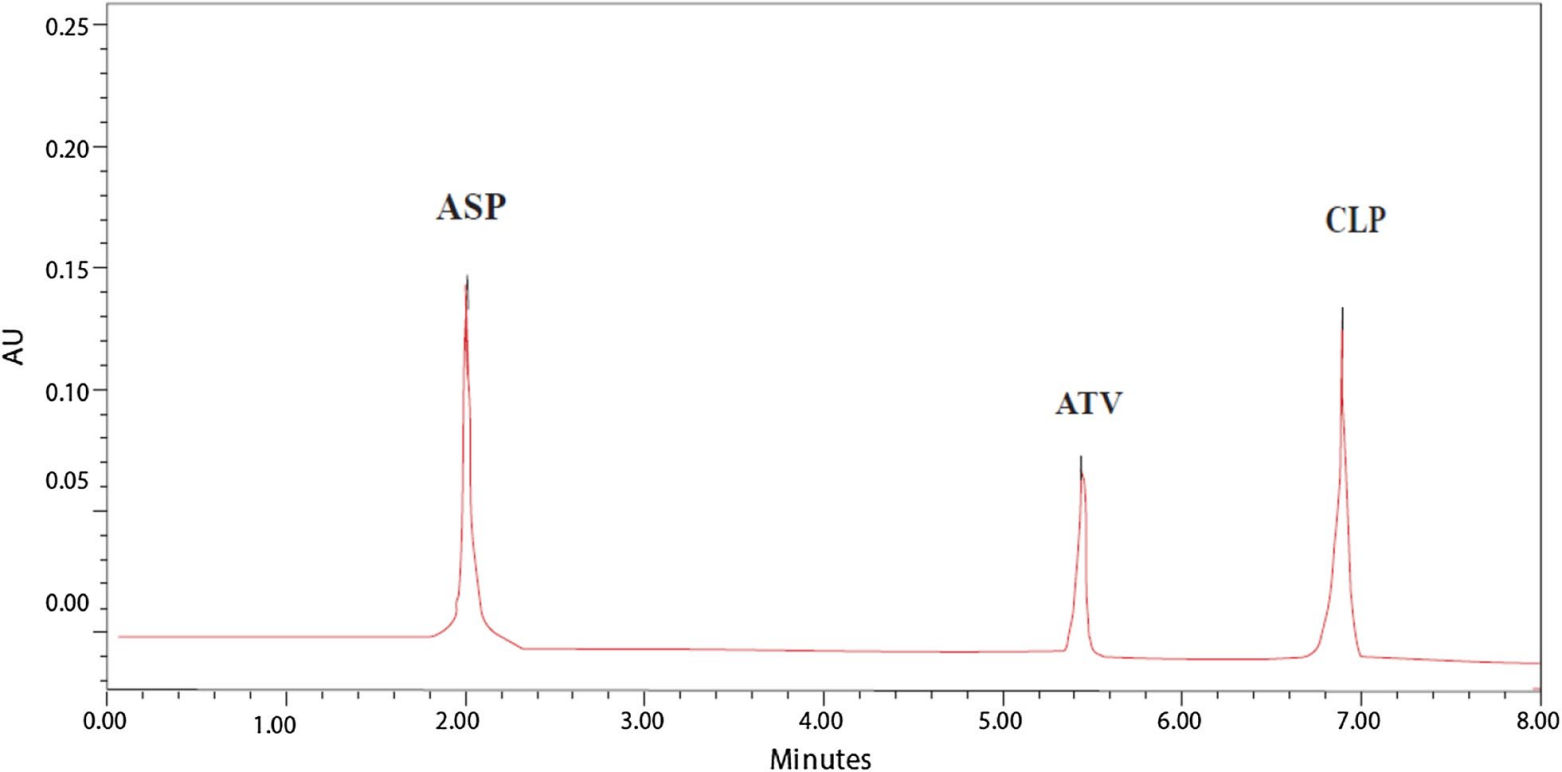
HPLC chromatogram of ASP (7.5 μg/mL), ATV (1 μg/mL) and CLP (7.5 μg/mL) in their FDC tablets

**Fig. 8:**
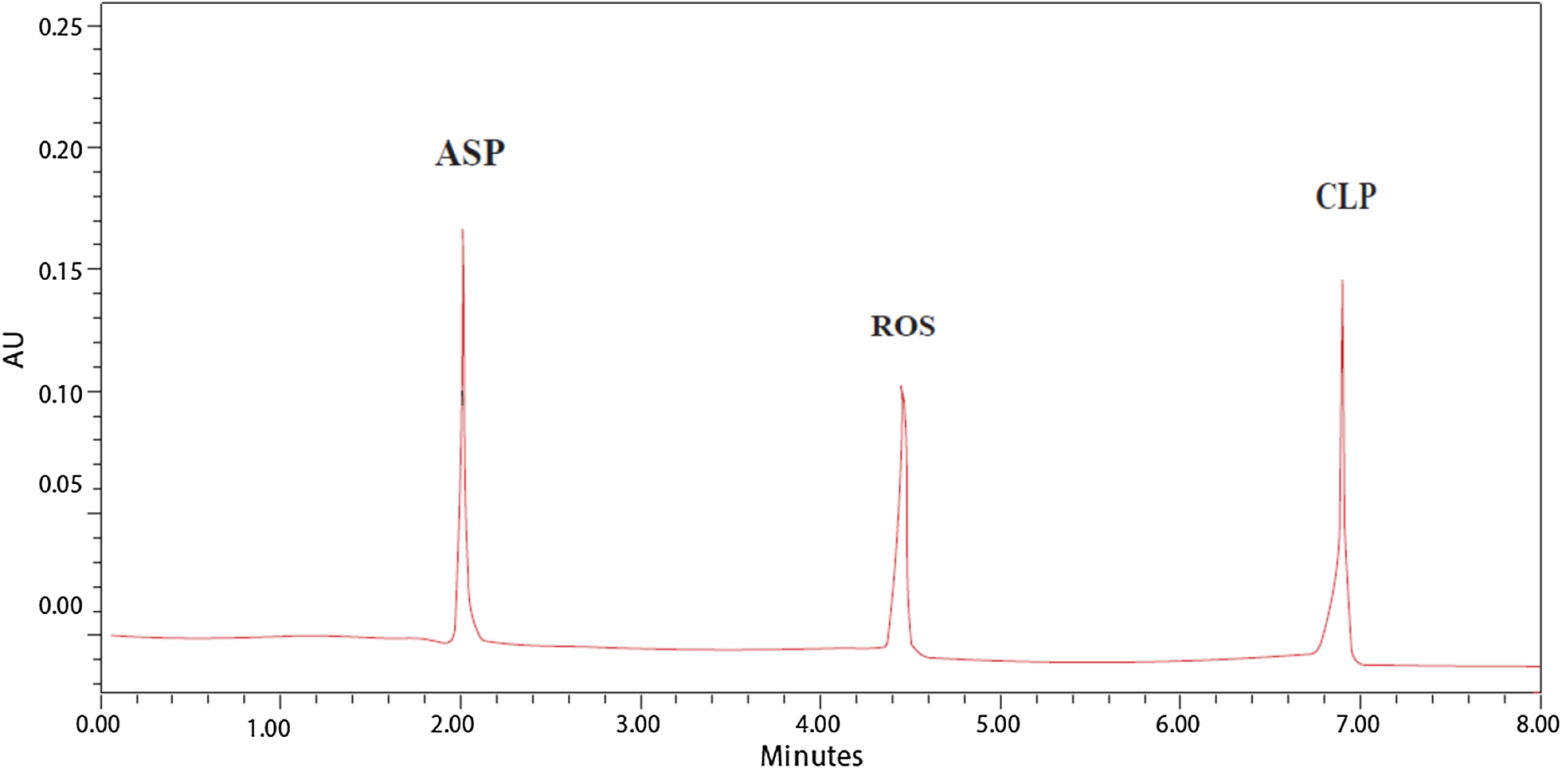
HPLC chromatogram of ASP (7.5 μg/mL), ROS (1 μg/mL) and CLP (7.5 μg/mL) in their FDC tablets

- (a) Linearity
  In this study, six concentrations were selected. Each concentration was analyzed three times. High regression coefficients were attained, demonstrating the calibration curve’s good linearity. The analytical data of the calibration curve including standard deviations for the slope (S_b_) and that of the intercept (S_a_) are summarized in (Table 4).
- (b) Accuracy
  To show the accuracy of the results in a laboratory-prepared mixture, percent recoveries of six different concentrations of ASP, CLP and ATV or ASP, CLP and ROS were calculated and injected in triplicates. The findings, including the recovery mean and standard deviation, are shown in (Tables 5, 6).
- (c) Precision
  The repeatability (the intra-day) of the method was assessed by six determinations for each of the three concentrations of *ASP* (1.5, 15 and 25 µg/mL), *ROS* (0.1, 1 and 10 µg/mL), *ATV* (0.1, 1 and 10 µg/mL) and *CLP* (0.75, 7.5 and 35 µg/mL) and was expressed in terms of % RSD. It was found to be less than 1% for the three concentrations. For intermediate precision (the inter-day), all experiments conducted in repeatability were repeated in three different days to evaluate day to day ruggedness. Results for the determination of repeatability and intermediate precision are represented in (Table 4).
- (d) Specificity
  The capacity of an analytical technique to predict the analyte response in the presence of interferences is known as specificity. By assessing ASP, CLP and ATV or ASP, CLP and ROS in laboratory prepared mixtures containing different ratios of the intact drugs and ASP degradation product. Figure 6 demonstrated high resolution and the lack of any interfering degradation products. Furthermore, the chromatogram of the pharmaceutical dosage forms samples was checked for the appearance of any extra peaks, Figs. 7, 8. The chromatogram of ASP, CLP, ATV and ROS in the sample solutions matched those attained by the standard solution. Additionally, good results were obtained for the determination of the cited drugs in dosage forms, (Table 4). These results prove the specificity of the developed method.
- (e) Limit of detection and limit of quantitation
  Results are provided in (Table 4) for the estimation of the limits of detection (LOD) and quantification (LOQ) based on the standard deviation of the response and the slope of the regression equation.
- (f) System suitability tests
  System suitability evaluations are essential for improving the conditions of the proposed technique in liquid chromatographic methods [51]. They are used to check the accuracy and repeatability of the analysis performed. These tests parameters are tailing of chromatographic peak, column efficiency (number of theoretical plates), repeatability as percentage relative standard deviation (%RSD) of peak area for six injections of a solution of a 10 µg/mL and reproducibility of retention as %RSD of retention time. The outcomes of these tests for the created technique are presented in (Table 7).

**Table 4:** Validation parameters and results obtained by the proposed HPLC–DAD method for the simultaneous determination of ASP, ROS, ATV and CLP

Item	ASP	ROS	ATV	CLP
Retention time (*t*_*R*_) (min)	1.27	4.45	5.55	6.90
Wavelength of detection (nm)	230	230	230	230
Range of linearity (μg/mL)	0.05–50	0.05–50	0.05–50	0.05–50
Regression equation	AUP^a^ 210 nm = 1.1345 C_ASP_ + 0.3893	AUP 210 nm = 1.0273 C_ROS_ + 0.0251	AUP 210 nm = 1.8642 C_ATV_ + 0.1093	AUP 210 nm = 0.7693 C_CLP_ + 0.0067
Regression coefficient (r^2^)	0.9997	0.9997	0.9999	1
LOD (μg/mL)^b^	0.002	0.009	0.010	0.012
LOQ (μg/mL)^c^	0.006	0.027	0.032	0.037
Standard deviation of slope (S_b_)	0.00777	0.00680	0.00668	0.00139
Standard deviation of the intercept (S_a_)	0.18486	0.16199	0.15899	0.03310
Confidence limit of the slope	1.1345 ± 0.0183	1.0273 ± 0.0161	1.8642 ± 0.0158	0.7693 ± 0.0033
Confidence limit of the intercept	0.3893 ± 0.436	0.0251 ± 0.382	0.1093 ± 0.375	0.0067 ± 0.078
Standard error of estimation	0.36722	0.32179	0.31583	0.06574
^d^Intra-day % RSD	0.002–0.408	0.027–0.669	0.121–0.309	0.053–0.481
^e^Inter-day % RSD	0.296–0.512	0.222–0.595	0.291–0.415	0.115–0.813
Drug in dosage form	99.02 ± 0.112	99.56 ± 0.222	100.02 ± 0.142	100.14 ± 0.044
Drug added	99.45 ± 0.244	99.73 ± 0.347	98.99 ± 0.025	99.36 ± 0.012

^a^AUP: Area Under Peak*10^–5^

^b^LOD: 3.3*SD/slope

^c^LOQ: 10*SD/slope

^d^The intra-day (*n* = 3), average of three concentrations of *ASP* (1.5, 15 and 25 μg/mL), *ROS* (0.1, 1 and 10 μg/mL), *ATV* (0.1, 1 and 10 μg/mL) and *CLP* (0.75, 7.5 and 35 μg/mL) repeated three times within the day

^e^The inter-day (*n* = 3), average of three concentrations of *ASP* (1.5, 15 and 25 μg/mL), *ROS* (0.1, 1 and 10 μg/mL), *ATV* (0.1, 1 and 10 μg/mL) and *CLP* (0.75, 7.5 and 35 μg/mL) repeated three times within the day

**Table 5:** Determination of ASP, ATV and CLP in laboratory prepared mixtures using the developed HPLC–DAD method

Taken (μg/mL)			AUP			Found (μg/mL)			Recovery %		
ASP	ATV	CLP	ASP	ATV	CLP	ASP	ATV	CLP	ASP	ATV	CLP
1.5	0.1	0.75	2.083	0.295	0.586	1.493	0.100	0.753	99.53	100.00	100.40
5	0.5	5	5.991	1.031	3.849	4.938	0.494	4.995	98.76	98.80	99.90
7.5	1	7.5	8.876	1.96	5.789	7.481	0.993	7.516	99.75	99.30	100.21
15	2	7.5	17.255	3.805	5.775	14.866	1.982	7.498	99.11	99.10	99.97
25	5	25	28.593	9.323	19.269	24.860	4.942	25.039	99.44	98.84	100.16
50	10	35	57.128	18.567	26.963	50.012	9.901	35.040	100.02	99.01	100.11
								Mean	99.44	99.18	100.13
								± SD	0.451	0.443	0.178
								± RSD%	0.454	0.447	0.178

**Table 6:** Determination of ASP, ROS and CLP in laboratory prepared mixtures using the developed HPLC–DAD method

Taken (μg/mL)			AUP			Found (μg/mL)			Recovery %		
ASP	ROS	CLP	ASP	ROS	CLP	ASP	ROS	CLP	ASP	ROS	CLP
1.5	0.1	0.75	2.077	0.128	0.583	1.488	0.100	0.749	99.20	100.00	99.87
5	0.5	5	6.007	0.533	3.831	4.952	0.494	4.971	99.04	98.80	99.42
7.5	1	7.5	8.873	1.048	5.752	7.478	0.996	7.468	99.71	99.60	99.57
15	2	7.5	17.278	2.089	5.752	14.886	2.009	7.468	99.24	100.45	99.57
25	5	25	28.722	5.125	19.21	24.974	4.964	24.962	99.90	99.28	99.85
50	10	35	57.100	10.334	26.953	49.987	10.035	35.027	99.97	100.35	100.08
								Mean	99.51	99.75	99.73
								± SD	0.398	0.642	0.244
								± RSD%	0.400	0.643	0.245

**Table 7:** System suitability tests of the developed HPLC–DAD method for the simultaneous determination of ASP, ROS, ATV and CLP

Parameter	ASP	ROS	ATV	CLP	Reference value
N	2974	58,620	23,660	67,625	The higher the value, the more efficient the column is
R		25.8	8.8	10.1	> 2
T	1.20	0.95	1.05	0.82	≤ 2
K′	1.02	3.45	4.55	5.90	1–10
α		12.95	1.32	1.30	≥ 1

*N* number of theoretical plates, *R* resolution factor, *T* tailing factor, *K′* capacity factor, *α* selectivity factor

### Green profile evaluation

The most widely used of the greenness assessment tools are NEMI, ESA, GAPI, and the recently developed tool in 2020; AGREE because they can be used with the majority of analytical techniques. In order to evaluate the green profile of the current study and also to compare it with the previously reported methods for the determination of ASP, CLP, ATV and ROS, those four tools were utilized.

- (a) National environmental methods index (NEMI)
  NEMI was the original method developed for method selection and comparing the analytical parameters of the analytical method. Although it is a simple one and it is basic enough for consumers to understand the process by a quick look, but the information gathered is quite general and it cannot be categorized as semi-quantitative. it is evident from a comparison of the pictograms acquired using the created method and those that were previously reported that there is no difference between them, even though their detailed variability as will be reviewed later in the following metrics, (Tables 8, 9).
- (b) Eco-scale assessment (ESA)
  Compared to NEMI, this tool considers more information about the analytical processes [37]. It depends on the calculations used to determine the penalty points that were allocated for the procedure based on the types of reagents and solvents used, workplace dangers, the amount of energy utilized by the equipment employed, and the quantity of waste generated throughout the entire process. A figure is produced by subtracting the total penalty points allocated for the procedure from 100 as the outcome of ESA. The analytical method is greener as it gets closer to 100, with 100 representing the optimum green analytical approach. The proposed approach received a remarkable score of 90 when it was evaluated by the ESA, defining it as an excellent green method, which was the primary goal of the current study. Despite the fact that four of the reported methods [12, 15, 22, 24] received the same ESA score as the proposed one, the developed method has turned out to be the most environmentally friendly among all of the reported methods after thorough investigation of its details using AGREE, as will be discussed later. All ESA scores of the developed method and the reported ones have been described in detail in (Tables 8, 9).
- (c) Green analytical procedure index (GAPI)
  The third discussed tool is GAPI which is based on a pictogram made up of five pentagrams. Each pentagram depicts the impact on the environment of a certain step in the analytical process. Three colors—green, yellow, and red—denote the degree of the environmental damage. GAPI provides the unique opportunity to combine the benefits of NEMI and ESA as it provides a succinct summary and in-depth analysis of how environmentally friendly particular steps of the analytical process are [32]. The created method and the reported ones are colored in the same way in the first pentagram, which has four fields and is connected to sampling. The second pentagram, which has just one field, is associated with the type of technique. The developed approach and the ones that have been reported are highlighted in yellow since they all call for easy sample preparation steps. Every method in this pentagram has a circle in the center since they are all quantitative procedures. The third pentagram has three fields including extraction scale, used reagents, and additional treatments. While the fourth pentagram discusses the quantity of solvents and the associated health and safety issues. Since the amount of solvents used in all the methods fall in the range of 10–100 mL, this field is colored yellow in all methods. In conclusion, all approaches are colored the same with regard to the third and fourth pentagrams. The instrument’s energy usage, workplace risks, trash generation, and handling are all addressed in the fifth pentagram. The methods [12, 14] are considered the least green in the fifth pentagram. Where the field number 14 is colored red because the quantity of waste produced is larger than 10 mL, (Tables 8, 9).

**Table 8:** ESA, NEMI, GAPI and AGREE tools for greenness assessment of recently published chromatographic methods and the developed method for simultaneous determination of ASP, CLP and ATV

Chromatographic method	ESA			NEMI	GAPI	AGREE
The developed method	Reagents		Penalty Points			
		Acetonitrile	4			
		water	0			
		phosphate buffer	0			
		Phosphoric acid	2			
	Σ		6			
	HPLC/UPLC		1			
	Waste		3			
	Occupational hazards		0			
	Σ		4			
	Total penalty points		10			
	ESA score		90			
[11]	Reagents	Acetonitrile	4			
		Methanol	6			
		Triethylamine	6			
		Phosphoric acid	2			
	Σ		18			
	HPLC/UPLC		1			
	Waste		3			
	Occupational hazards		0			
	Σ		4			
	Total penalty points		22			
	ESA score		78			
[12]	Reagents	Acetonitrile	4			
		Phosphate buffer	0			
		Phosphoric acid	2			
	Σ		6			
	HPLC/UPLC		1			
	Waste		3			
	Occupational hazards		0			
	Σ		4			
	Total penalty points		10			
	ESA score		90			
[13]	Reagents	Acetonitrile	4			
		Methanol	6			
		Phosphate buffer	0			
		Phosphoric acid	2			
	Σ		12			
	HPLC/UPLC		1			
	Waste		3			
	Occupational hazards		0			
	Σ		4			
	Total penalty points		16			
	ESA score		84			
[14]	Reagents	Acetonitrile	4			
		Methanol	6			
		Water	0			
		Phosphoric acid	2			
	Σ		12			
	HPLC/UPLC		1			
	Waste		3			
	Occupational hazards		0			
	Σ		4			
	Total penalty points		16			
	ESA score		84			
[15]	Reagents	Acetonitrile	4			
		Phosphate buffer	0			
		Phosphoric acid	2			
	Σ		6			
	HPLC/UPLC		1			
	Waste		3			
	Occupational hazards		0			
	Σ		4			
	Total penalty points		10			
	ESA score		90

**Table 9:** ESA, NEMI, GAPI and AGREE tools for greenness assessment of recently published chromatographic methods and the developed method for simultaneous determination of ASP, CLP and ROS

Chromatographic method	ESA			NEMI	GAPI	AGREE
The developed method	Reagents		Penalty Points			
		Acetonitrile	4			
		water	0			
		phosphate buffer	0			
		Phosphoric acid	2			
	Σ		6			
	HPLC/UPLC		1			
	Waste		3			
	Occupational hazards		0			
	Σ		4			
	Total penalty points		10			
	ESA score		90			
[21]	Reagents	Acetonitrile	4			
		water	0			
		phosphate buffer	0			
		acetic acid	2			
	Σ		6			
	HPLC/UPLC		1			
	Waste		3			
	Occupational hazards		0			
	Σ		4			
	Total penalty points		10			
	ESA score		90			
[22]	Reagents	Methanol	6			
		water	0			
		Phosphoric acid	2			
	Σ		8			
	HPLC/UPLC		1			
	Waste		3			
	Occupational hazards		0			
	Σ		4			
	Total penalty points		12			
	ESA score		88			
[23]	Reagents	Acetonitrile	4			
		water	0			
		phosphate buffer	0			
		Phosphoric acid	2			
	Σ		6			
	HPLC/UPLC		1			
	Waste		3			
	Occupational hazards		0			
	Σ		4			
	Total penalty points		10			
	ESA score		90			

### Analytical greenness metric (AGREE)

AGREE, which was released in 2020 [39], is the most recent greenness assessment instrument to be developed. AGREE is based on the 12 GAC principles and is divided into 12 segments, each of which is colored according to how much green it includes on a scale from 0 to 1, with 1 being the most green and 0 the least (red color). The developed approach received the highest rating of 0.54. Contrarily, the reported approach [12] has the lowest score (0.46) due to the use of a considerable amount of organic solvent and the lengthy sample extraction process, as well as the lengthy run duration of 20 min. The created method's and reported methods' acquired pictograms of AGREE scoring are shown in (Tables 8, 9). Due to its minimal waste production, reasonable run time, and higher number of analytes per run, the developed technique has the advantage over the ones that have been reported in terms of the AGREE score. While ESA score could be the same for different methods, AGREE score could be different for the same methods. That is a fact since AGREE score considers so many details which are not considered in ESA. For example, the number of analytes per single run, the threats which related to the application of reagents, in which case material safety data sheets provide a clear indication.

After looking into the developed method's green profile, it could be said that it is an environmentally friendly one that has been rated as an exceptional green method by ESA with an ESA score of 90. Also, it received the highest AGREE score of 0.54 among the compared methods.

## Conclusion

A statistically based Box-Behnken design was employed during method optimization approach to attain optimal peak shape and resolution with minimal experimental trials, for the development of a RP-HPLC method for the determination of ASP, CLP, ATV and ROS in presence of the ASP degradation product in bulk powder and in pharmaceutical dosage forms. Greenness assessment approaches were utilized for analysis of the greenness profile of the proposed method and to perform a detailed greenness comparison between the developed method and some of the reported methods for the determination of the investigated drugs. The developed method was found to be an eco-friendly method with the highest AGREE score among the compared methods. The suggested technique was determined to be effective, quick, accurate, precise, and robust and may be utilized for the routine analysis of ASP, CLP, ATV and ROS in pure powder or FDC tablets. A further modification is intended for the current study to be able for the determination of the cited drugs in plasma.

## Data Availability

All data generated or analyzed during this study are included in this published article.
